# Separation of color channels from conventional colonoscopy images improves deep neural network detection of polyps

**DOI:** 10.1117/1.JBO.26.1.015001

**Published:** 2021-01-13

**Authors:** Lily L. Lai, Andrew Blakely, Marta Invernizzi, James Lin, Trilokesh Kidambi, Kurt A. Melstrom, Kevin Yu, Thomas Lu

**Affiliations:** a City of Hope, Department of Surgery, Duarte, California, United States; b National Cancer Institute, National Institutes of Health Campus, Department of Surgery, Bethesda, Maryland, United States; c City of Hope, Division of Gastroenterology, Duarte, California, United States; d Jet Propulsion Labarotory, Pasadena, California, United States

**Keywords:** artificial intelligence algorithms, deep learning, polyp discrimination, colorectal cancer, narrow-band imaging, color channel separation

## Abstract

**Significance**: Colorectal cancer incidence has decreased largely due to detection and removal of polyps. Computer-aided diagnosis development may improve on polyp detection and discrimination.

**Aim**: To advance detection and discrimination using currently available commercial colonoscopy systems, we developed a deep neural network (DNN) separating the color channels from images acquired under narrow-band imaging (NBI) and white-light endoscopy (WLE).

**Approach**: Images of normal colon mucosa and polyps from colonoscopies were studied. Each color image was extracted based on the color channel: red/green/blue. A multilayer DNN was trained using one-channel, two-channel, and full-color images. The trained DNN was then tested for performance in detection of polyps.

**Results**: The DNN performed better using full-colored NBI over WLE images in the detection of polyps. Furthermore, the DNN performed better using the two-channel red + green images when compared to full-color WLE images.

**Conclusions**: The separation of color channels from full-color NBI and WLE images taken from commercially available colonoscopes may improve the ability of the DNN to detect and discriminate polyps. Further studies are needed to better determine the color channels and combination of channels to include and exclude in DNN development for clinical use.

## 1 Background

Colorectal cancer (CRC) is the second leading cause of cancer and third leading cause of cancer deaths in the US. Over the last five years, there has been a steady decline in the incidence of CRC.^1^ Most of this is attributed to ability to prevent CRC through the use of screening colonoscopies. By identifying polyps, the precursors of CRC, and removing the polyps at the same time, colonoscopy use has been shown to decrease the incidence of CRC by 90%^2^ and risk of CRC-related death by 53%.^3^

However, variability in the ability to identify polyps from normal mucosa and to differentiate those polyps as cancerous versus non-cancerous remains challenging. Image analysis using machine learning has emerged, with the potential to improve on polyp detection and classification.^4^^,^^5^ Universal application and clinical adoption have been limited by the need for “training” the machine learning algorithm, which requires hand-crafted feature extraction and a considerable hand-engineering of imaging features to generate polyp classification.^6^^,^^7^ Furthermore, other technology such as high-magnification endoscopy and laser-induced fluorescence spectroscopy may be required for better image feature extraction, which is not routinely performed in clinical practice.^5^^,^^8^

More recently, artificial intelligence (AI), in particular deep learning (DL) and resultant deep neural networks (DNN), has enabled more detailed image analysis by the autonomous extraction of relevant image features, transforming the field of pattern recognition for complex images in the colonoscopy detection and discrimination of polyps.^9^^–^^11^ We hypothesize that development of a DNN from already available images in full color and separated by color channels, taken during routine colonoscopy using commercially available scopes, will improve on polyp identification and discrimination. In this paper, we describe our initial development and performance of a DNN in the detection of colonic polyps.

## 2 Methods

### 2.1 Clinical Study

The clinical study was completed after institutional approval and conducted under the supervision of the institutional regulatory committees. Sixteen patients scheduled to undergo screening or surveillance colonoscopy were accrued to the clinical study. Informed consent was obtained from all subjects.

### 2.2 Clinical Study Workflow

Once the patient was sedated per colonoscopy protocol, the colonoscope was inserted. Under insufflation, the scope was advanced to the ileocecal valve. The colonoscope was slowly withdrawn to evaluate the epithelial surface. Polyps identified and determined to require biopsy, based on the clinician’s judgment, were photographed using white-light endoscopy (WLE) and under narrow-band imaging (NBI). The WLE images are RGB images because they have three channels: red (wavelength range: 664 to 680 nm); green (wavelength range: 534 to 545 nm); and blue (wavelength range: 420 to 440 nm). The NBI image contains two narrow color filters centered at 415 and 540 nm, respectively. Each polyp was imaged 12 times, six times under WLE, and six times under NBI. WLE and NBI images of the normal mucosa along the length of the colon were obtained for comparison. The polyps removed were evaluated through the pathology department and clinical treatment was per standard-of-care.

The colonoscopic images were stored on the hard drive of the commercial colonoscopy unit in Joint Photographic Experts Group format. The image resolution is 96 dpi and the image size is 720×480  pixels.

### 2.3 Materials/Equipment

The colonoscopy equipment and processing unit were manufactured by Olympus^®^ America Inc. (Southborough, Massachusetts), Three colonovideoscope models were used: EVIS EXERA III (CF-HQ190L/I—Sn: 2876181), EVIS EXERA II (PCF-H180AL/I—Sn: 2109101), and EVIS EXERA III (PCF-H190DL/I—Sn: 2840944, 2840948). The Imaging System Video Processor model used is EVIS EXERA III CV-190.

### 2.4 DNN Development

**Fig. 1:**
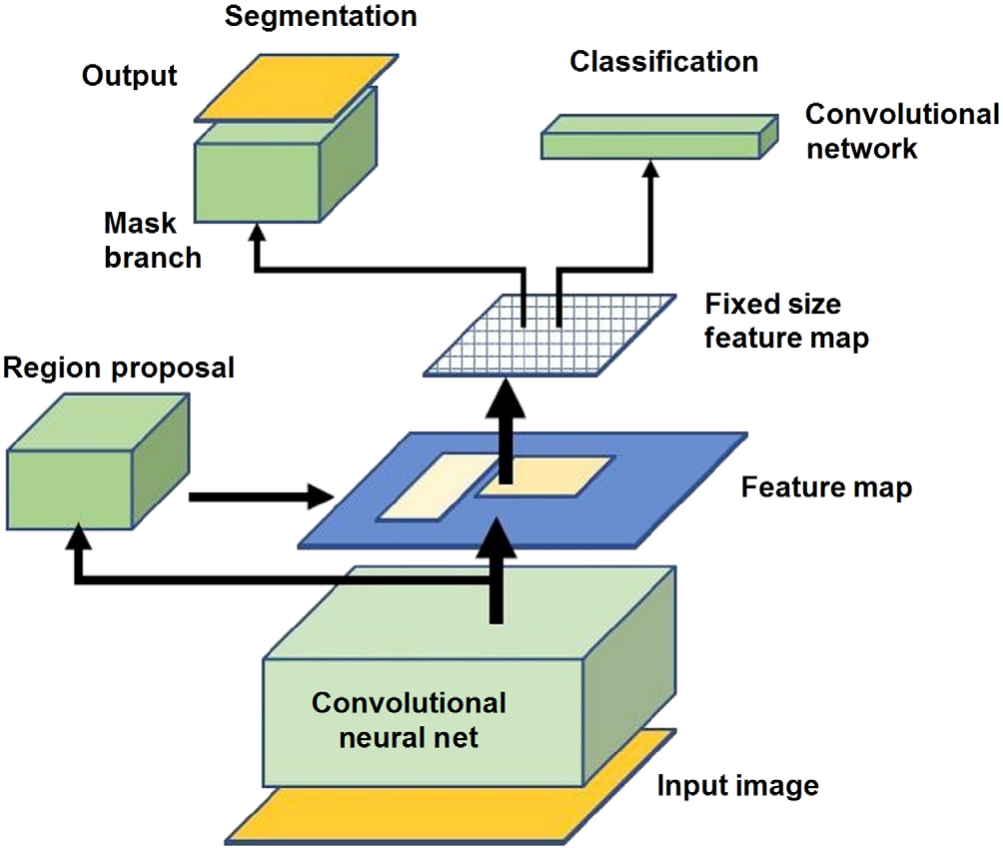
Schematic of Mask-RCNN.

For the polyp detection and segmentation, a DNN model, mask region-based convolutional neural network (Mask-RCNN), was used to identify and segment or delineate objects within images.^12^ This network was used due to its performance and ability to identify objects and generate precise segmentation masks. In instance segmentation, a mask is generated around each object along with a bounding box. From this approach, information on the location, pixel boundary, and quantities of the objects can be obtained (Fig. 1).

In this research, a special implementation of Mask-RCNN was used for training and testing on the NBI images and compared with the WLE images.^9^ Mask-RCNN expands upon Faster-RCNN by adding an additional mask branch to the existing bounding box branch. Mask-RCNN outputs a class label, bounding box, and precise boundary mask for each object. Figure 1 shows the network architecture of a Mask-RCNN, which comprises of the convolutional neural network (CNN), region proposal network, bounding box head, and mask head.

Multiple training sessions using the Mask-RCNN were conducted using full-color NBI and WLE images, individual color channel images, and combination of the two-color channel images. The best performing DNN in full color, each color channel, and combined color channels were saved and tested using separate testing image sets that were not in the training data. Accuracy, sensitivity, specificity, and precision were calculated for each DNN as measures of performance.

## 3 Results

Over a 12-month period, 16 patients were accrued into the study. Patient demographics and clinical characteristics are given in Table 1.

**Table 1:** Demographic and clinical data.

Record ID	Age	Gender	Number of polyps	Polyp description			
				Anatomic location	Size (mm)	Polyp type	Diagnosis
1	60	M	0				
2	64	M	3	Right colon	3	Tubular	Tubular adenoma
				Right colon	7	Tubular	Tubular adenoma
				Left colon	8	Tubular	Tubular adenoma
3	74	F	3	Right colon	8	Tubulovillous	Tubulovillous adenoma
				Right colon	3	Serrated	Sessile serrated adenoma
				Left colon	8	Tubular	Tubular adenoma
4	51	M	2	Sigmoid	5	Hyperplastic	Hyperplastic polyp
				Rectum	10	Tubulovillous	Tubulovillous adenoma
5	70	M	1	Right colon	2	Tubular	Tubular adenoma
6	51	M	0	—	—	—	—
7	52	F	0	—	—	—	—
8	62	F	1	Rectum	2	—	Adenoma
9	80	M	0	—	—	—	—
10	59	F	1	Right colon	3	Tubular	Tubular adenoma
11	65	F	2	Right colon	3	Tubular	Tubular adenoma
				Rectum	3	Tubular	Tubular adenoma
12	55	M	2	Sigmoid	14	Tubular	Tubular adenoma
				Sigmoid	5	Tubular	Tubular adenoma
13	55	F	0	—	—	—	—
14	73	F	0	—	—	—	—
15	67	F	0	—	—	—	—
16	65	F	0	—	—	—	—

The mean age of the patients was 65 (±14.5) years, and 56% (9/16) of the patients were female. The indication for the colonoscopy was screening in 12 patients and surveillance in four patients with a history of colon cancer. The colonoscopies were completed by three investigators (James Lin, Kurt Melstrom, and Trilokesh Kidambi). Histopathologic data on the imaged polyps, which were then removed, are given in Table 1. Images were taken of normal colon and identified polyps. There was a total of 15 polyps in the 16 patients (Table 1). Of the 15 polyps, nine lesions were ≤5  mm; four lesions were 6 to 9 mm; and two lesions were ≥10  mm. The polyps were tubular adenomas (n=11), tubulovillous adenomas (n=2), sessile serrated (n=1), and hyperplastic (n=1).

### 3.1 Training DNN for NBI Image Recognition

**Fig. 2:**
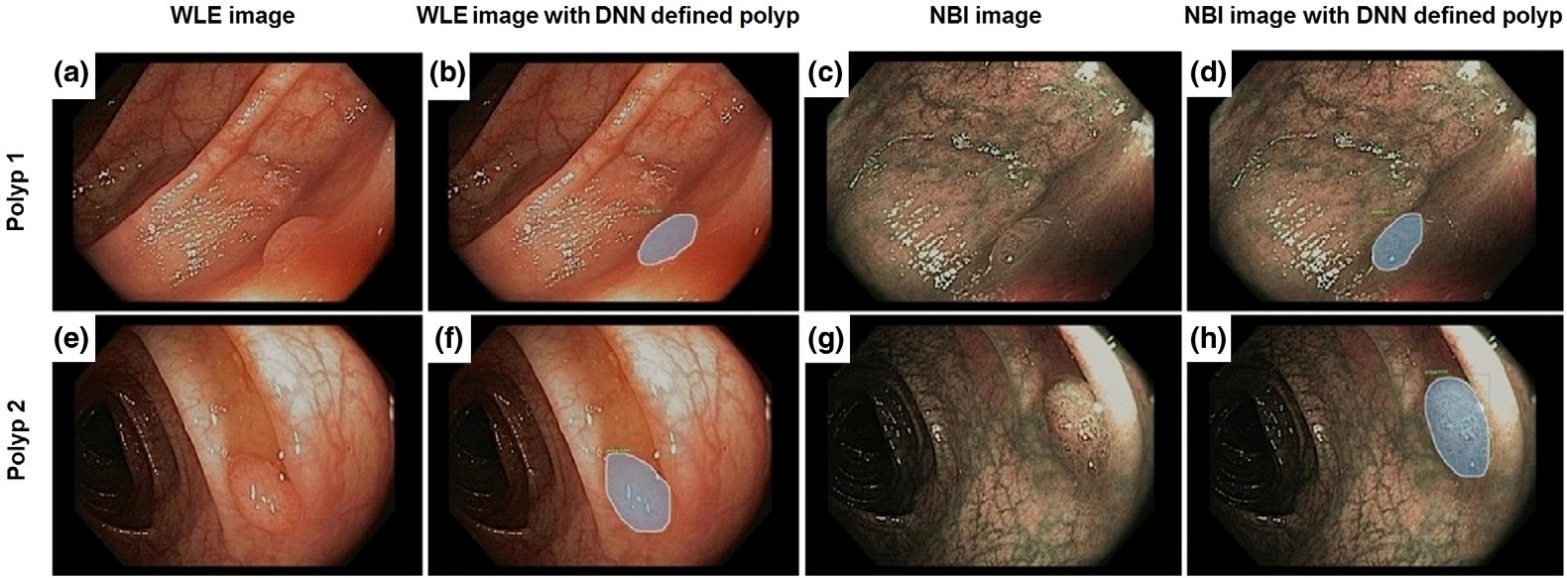
(a), (b), (e), and (f) DNN recognition of polyps in WLE and in (c), (d), (g), and (h) NBI. (a) and (e) WLE images of polyps. (b) and (f) DNN recognition of the polyps. The gray shading demonstrates the DNN detection of the polyp and the boundaries of the polyp. (c) and (g) NBI images of polyps. (d) and (h) DNN recognition of the polyps. The gray shading demonstrates the DNN detection of the polyp and the boundaries of the polyp.

A training image set of 50 images (20 negatives, 30 positives) of NBI images were selected for training a DNN based on the Mask-RCNN model described above. The training images were carefully selected so that the network learns the variance of the polyps and background but not to be confused by ambiguities. The DNN was trained on a GoogleCloud server with graphics processing units (GPUs). The learning rate is 0.0025. The DNN was trained for a total of 1500 iterations. Examples of NBI images are provided (Fig. 2). Training images both include images with or without a polyp as well as a segmentation mask around the boundaries of the polyp.

### 3.2 Training DNN for WLE Image Recognition

A training image set of 74 WLE images (34 negatives, 40 positives) were used for training a DNN. Similarly, the DNN was trained on a GoogleCloud server with GPUs. The learning rate is 0.0025. The DNN was trained for a total of 1500 iterations. Examples of WLE images are provided (Fig. 2).

### 3.3 Training DNN on Separate Color Channels for NBI and WLE Images

**Fig. 3:**
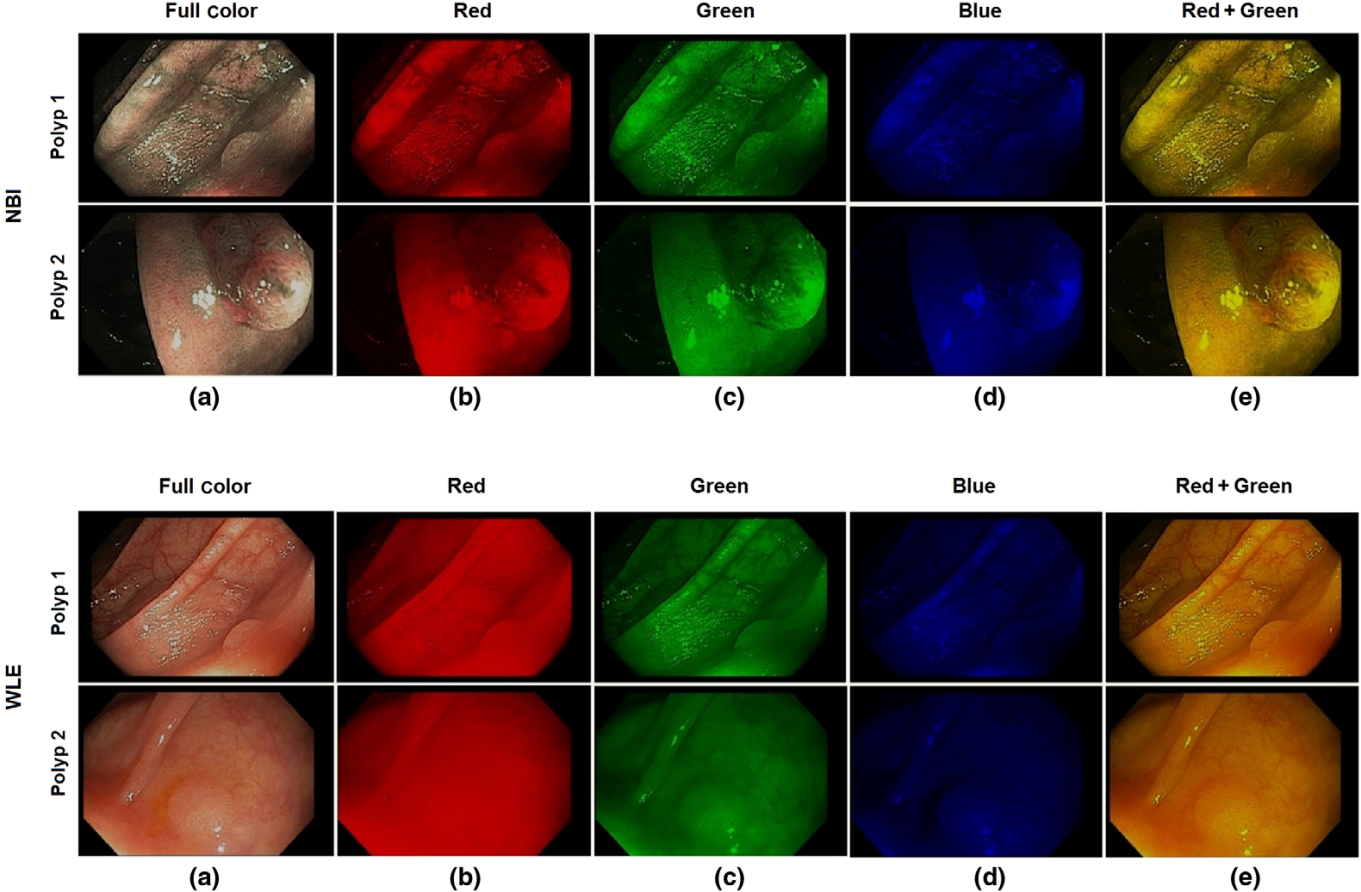
Examples of NBI and WLE images: (a) full color, (b) red channel, (c) green channel, (d) blue channel, and (e) red + green channel.

In this experiment, a total of 112 NBI were divided into 93 training images and 19 testing images. A total of 150 WLE images were divided into 127 training and 23 testing images. The NBI and WLE images are color images with three channels: red/green/blue (RGB). The resolution of the images is 480×720  pixels. Each pixel has an RGB value, such that an image is expressed as a 480×720×3 array. A single 480×720×1 channel was extracted corresponding to each color, and the other two channels were padded with zeros so that only the data from each color channel is retained. In this way, the original size of the image is retained. For a combination of two color channels, two of the three channels were retained, and the third channel was padded with zeros. Examples of full color, single and two-channels NBI, and WLE images are shown in Fig. 3.

A 101-layer DNN, as described in Mask-RCNN Model section, was trained around 3000 epochs with the training set to learn the images features and to thereby distinguish the polyps from normal tissue in both NBI and WLE images. After multiple training sessions to train the full color, single channel, and combination channel DNNs on NBI and WLE images, the best performing DNNs were tested with separate sets of testing images. The testing results of NBI and WLE images are shown in Table 2.

**Table 2:** Performance of the DNN polyp detection in NBI and WLE images.

NBI image testing (Total TP = 14 Total TN = 5)					
	Full color	Red	Green	Blue	Red + green
True positive (TP)	13	12	12	10	13
True negative (TN)	5	5	5	5	5
Sensitivity TP/(TP + FN) (%)	93	86	86	71	93
Specificity: TN/(TN + FP) (%)	100	100	100	100	100
Precision: TP/(TP + FP) (%)	100	100	100	100	100
**Accuracy: (TP + TN)/(TP + TN + FP + FN) (%)**	**95**	**89**	**89**	**79**	**95**
WLE image testing (Total TP = 15 Total TN = 8)					
	Full color	Red	Green	Blue	Red + green
True Positive (TP)	9	9	7	6	13
True Negative (TN)	8	8	7	7	8
Sensitivity TP/(TP + FN) (%)	60	60	47	40	87
Specificity: TN/(TN + FP) (%)	100	100	87	87	100
Precision: TP/(TP + FP) (%)	100	100	87	86	100
**Accuracy: (TP + TN)/(TP + TN + FP + FN) (%)**	**74**	**74**	**61**	**57**	**91**

### 3.4 DNN Performance in Polyp Detection

For both the full-color and individual color channel, we classify whether the DNN has identified the presence or lack of a polyp within an image. The accuracy is determined by the percentage of correctly classified images. In the NBI testing, there were a total of 14 polyp images and five normal images. The DNN identified 13 out of 14 polyp images and five out of five normal images in the full-color test images with an accuracy of 95%. The DNN performed less well in the single red (accuracy 89%), green (accuracy 89%), and blue (accuracy 79%) channels. The DNN performed the worst in blue channel. There was no difference in DNN performance between the two-channel red + green (accuracy 95%) than in the three-channel full-color images (Table 2).

In the WLE testing, there were a total of 13 polyp images and eight normal images. The DNN identified nine out of 13 polyp images and eight out of eight normal images in the full-color test images with an accuracy of 74%. The DNN performed as well in single red (accuracy 74%), less well in green (accuracy 61%), and less well in blue (accuracy 57%) channels. The DNN performed the worst on the blue channel. The DNN performed better in the two-channel red + green images with an accuracy of 91% compared with the lower accuracy (74%) in the three-channel full-color WLE images (Table 2).

## 4 Discussion

Over the last two decades, there have been technology advances such as NBI and confocal microendoscopy to improve on polyp detection and discrimination.^13^ In addition, careful characterization of polyp features has led to classification schemes that better identify polyps with higher malignant potential requiring additional clinical management.^6^^,^^14^ Computer-aided diagnosis (CAD) and AI have been applied to colonoscopy images and videos with resultant near real-time ability to detect and differentiate types of polyps captured in still images as well as in video.^5^^,^^15^ These systems remain investigational, without direct implementation yet into the clinical workflow, but with great potential to improve on current detection and discrimination of polyps.

Our study furthers the work of other groups by applying an artificial neural network to enable detection of colonic polyps. The principle is to train a DL algorithm to extract features from the full color, single channel, and two-channel images of an endoscope to enhance the differences between the polyp and surrounding normal tissue in captured in colonoscopy images. The DL algorithm automatically selects the best combination of image components to enhance the contrast between normal and abnormal tissues. Then, the DL algorithm performs automatic detection and segmentation of the polyps in the full color, single channel, and two-channel color images. Traditional neural networks contain one to three hidden layers, whereas DL models can contain as many as 100 hidden layers. As a result, this type of model is often referred to as a DNN.^16^ Typically, DNN models learn classification tasks from labeled datasets of images through a process called feature extraction. The multi-layer nature of a DNN allows for each layer to encode distinctive features.^12^

Our DNN was trained on full-color NBI and WLE images. In addition, images obtained from separating the color channels into single-channel red, blue, or green or into two-channel red + green were used to train the DNN. Consistent with the results of other studies,^10^^,^^11^^,^^15^ the DNN was more accurate in detecting polyps when using full-color NBI images with an accuracy of 95% compared with an accuracy of 74% using full-color WLE images. Overall, the accuracy of DNN detection did not improve with the separation of the images to single color channels for either NBI or WLE images. Instead, the DNN was more accurate when testing with the two-channel red + green images than with full-color WLE images, arguing that the addition of the blue channel may mask distinguishing features used by the DNN to make discriminations and negatively contributes to the WLE polyp detection process. As such, the DNN was least accurate when tested with the single-channel blue WLE images (accuracy 57%).

The main drawback of DNN is the requirement for a large number of training samples. In this work, a DNN model was built and trained with a small number of polyps and normal mucosa images. However, the overall accuracy of the DNN, even with the small number of images so far studied, argues that further training will increase the ability of the DNN to accurately detect polyps. In addition, the ability to separate the images into single-channel images and two-channel images for testing and training increases the dataset available for the DNN to learn. Another weakness of this current DNN is the level of computational processing required. Each single training process currently requires 2 h in a dedicated CPU + GPU environment and is completed off-line in a server given the data processing demands.

To optimize certain parameters such as the learning rate, multiple time-consuming training sessions were required. However, prediction of new images, requiring about 200 ms per image for classification and 2 to 3 s with segmentation, is rather quick. For clinical implementation, a scanning mode can be developed in which the trained DNN classifies the image. When there is a positive classification of a polyp in an image, the DNN can then search for the specific location and segment the boundary of the polyp. With improved data processing capabilities and application of a trained DNN, DNN-enabled real-time automated detection and diagnosis of CRC polyps and cancer into the clinical workflow may soon be reality.

## 5 Conclusions

We have built a DNN and trained it with images taken from commercially available colonoscopies. The images of polyps and normal tissue from 16 patients undergoing standard screening and surveillance colonoscopies. Initial testing results using the DNN on full-color NBI and WLE images, single color channel, and two-channel color images have shown high accuracy in the detection of polyps. Overall, the accuracy was greater in the NBI images than in the WLE images. However, the DNN demonstrated improved performance when tested on two-channel red + green WLE images compared with full-color WLE images. Further development of the DNN on other color channel combinations and on multi-band filtering may improve on accuracy of detection and may enable polyp discrimination. Additional validation studies are needed to test and refine the DNN.
